# Large-Scale Phylogenetic Analysis Reveals a New Genetic Clade among Escherichia coli O26 Strains

**DOI:** 10.1128/spectrum.02525-21

**Published:** 2022-02-02

**Authors:** Jinzhao Long, Juna Geng, Yake Xu, Yuefei Jin, Haiyan Yang, Yuanlin Xi, Shuaiyin Chen, Guangcai Duan

**Affiliations:** a College of Public Health, Zhengzhou University, Zhengzhou, Henan, People’s Republic of China; b Henan Province Centers for Disease Control and Prevention, Zhengzhou, Henan, People’s Republic of China; c Henan Key Laboratory of Molecular Medicine, Zhengzhou University, Zhengzhou, Henan, People’s Republic of China; Johns Hopkins Hospital

**Keywords:** CRISPR typing, *Escherichia coli* O26, genome evolution, whole-genome analysis

## Abstract

Shiga toxin-producing Escherichia coli (STEC) O26 is the predominant non-O157 serogroup causing hemolytic uremic syndrome worldwide. Moreover, the serogroup is highly dynamic and harbors several pathogenic clones. Here, we investigated the phylogenetic relationship of STEC O26 at a global level based on 1,367 strains from 20 countries deposited in NCBI and Enterobase databases. The whole-genome-based analysis identified a new genetic clade, called ST29C4. The new clade was unique in terms of multilocus sequence type (ST29), CRISPR (group Ia), and dominant plasmid gene profile (*ehxA*+/*katP*-/*espP*-/*etpD*-). Moreover, the combination of multiple typing methods (core genome single nucleotide polymorphism [SNP] typing, CRISPR typing, and virulence genes analysis) demonstrated that this new lineage ST29C4 was in the intermediate phylogenetic position between ST29C3 and other non-ST29C3 strains. Besides, we observed that ST29C4 harbored extraintestinal pathogenic E. coli (ExPEC)-related virulence gene (VG), *tsh*, and STEC-associated VG, *stx2a*, suggesting the emergence of a hybrid pathogen. The ST29C4 strains also exhibited high similarity in *stx2a*-prophage and integrase with the O104:H4 strain, further demonstrating its potential risk to human health. Collectively, the large-scale phylogenetic analysis extends the understanding of the clonal structure of O26 strains and provides new insights for O26 strain microevolution.

**IMPORTANCE** Shiga toxin-producing Escherichia coli (STEC) O26 is the second prevalent STEC serogroup only to O157, which can cause a series of diseases ranging from mild diarrhea to life-threatening hemolytic uremic syndrome (HUS). The serogroup is highly diverse and multiple clones are characterized, including ST29C1-C3 and ST21C1-C2. However, the phylogenetic relationship of these clones remains fully unclear. In this study, we revealed a new genetic clade among O26 strains, ST29C4, which was unique in terms of CRISPR, multilocus sequence type (MLST), and plasmid gene profile (PGP). Moreover, the combination of multiple typing methods demonstrated that this new clone was located in the intermediate phylogenetic position between ST29C3 and other non-ST29C3 strains (i.e., ST29C1-C2 and ST21C1-C2). Overall, the large-scale phylogenetic analysis extends our current understanding of O26 microevolution.

## INTRODUCTION

Shiga toxin-producing Escherichia coli (STEC) is recognized as the important zoonotic pathogen transmitted to humans through food, water, and animals (1, 2). STEC infections can cause various human diseases ranging from mild diarrhea to bloody diarrhea and life-threatening hemolytic uremic syndrome (HUS) (3). Although the serogroup O157 is the most common serotype among human cases infected with STEC, the proportion of non-O157 serogroups has steadily been increasing during past years, especially serogroup O26 (4–6). A report from the European Union (EU) showed that the serogroup O26 accounted for 14.3% of the confirmed human cases infected with STEC in the EU in 2017 and was the second predominant STEC serotype associated with HUS (7). Until 2018, STEC O26 has become the most common cause of HUS cases instead of O157 in Europe (8). In addition to European regions, multiple outbreaks linked to STEC O26 have been reported in the USA (9), Japan (10), and South Africa (11), thereby demonstrating the global dissemination of this STEC serogroup. Furthermore, the severity of HUS caused by STEC O26 is comparable with those caused by STEC O157 (12, 13).

STEC O26 strains are highly dynamic and gene turnover is frequent due to the acquisition or loss of key genetic elements, such as *stx*-carrying phages, plasmid-borne genes (14). Indeed, several clones of STEC O26 have been reported since serotype O26:H11 was first identified as a cause of HUS (15–17). These O26 clones display genetic distinctions in terms of four aspects: (i) multilocus sequence type (MLST); (ii) *stx* allele that encodes Shiga toxin; (iii) plasmid gene profiles (PGP) that consist of four plasmid-borne genes (*exhA*, *katP*, *espP*, *etpD*); and (iv) clustered regularly interspaced short palindromic repeats (CRISPR) spacer contents. Before the 1990s, most O26:H11 strains isolated from clinical samples possessed *stx1a* alone or rarely together with *stx2a* (15). These strains belonged to a monophyletic clone by MLST analysis, ST21. Plasmid gene profiling demonstrated that they lacked only one of four PGP-related genes (*etpD*) (18). Moreover, accumulating data suggested that this clone was responsible for mild diarrheas and sporadic outbreaks (15).

Since the 1990s, the O26 strains belonging to another MLST type, ST29, have been frequently detected from clinical patients (18, 19). In contrast to ST21 strains, ST29 strains are remarkedly heterogeneous and contain multiple monophyletic lineages. In the mid-1990s, the first highly virulent ST29 clone (called “new European clone”) carrying *stx2a* alone was reported in Germany (15). Since then, it has rapidly disseminated throughout Europe and the New World (20). This new European clone has attracted great attention due to its close relationship with HUS outbreaks. It is characterized by the absence of two out of four plasmid-borne genes (*katP* and *espP*). Subsequently, a distinct ST29 clone (referred to as the “new French clone”) that was negative for all the PGP-related genes (*ehxA*-/*katP*-/*espP*-/*etpD*-) emerged in France (16). This clone carries either *stx2a* or *stx2d*, which is closely related to *stx*-negative attaching and effacing Escherichia coli (21). Moreover, a CRISPR-targeting qPCR assay SP_O26_E is developed to detect the new French clone. Recently, another ST29 clone that harbored only *stx2a* was identified in Japan (17). The Japanese clone is phylogenetically distant to the new European clone and the new French clone and harbors a unique PGP (*ehxA*+/*katP*-/*espP*+/*etpD*-).

The epidemic of these various O26 clones around the world has demonstrated the genetic diversity of STEC O26. So far, several studies have started to investigate the clonal structure of O26:H11 strains (9, 17). However, most of these investigations only collected the strains isolated from a specific region or biasedly focused on one aspect of genetic characterization (e.g., MLST, CRISPR, and PGP). With the development of high-throughput DNA sequencing technology and the joint efforts of international researchers, a large number of genome sequences have been delivered to a public database (22). This provides an opportunity to assess the phylogenetic relationship of O26 strains from multiple geographical areas and to obtain a global overview of strain diversity. In this study, we conducted a whole-genome (WG)-based phylogeny based on 1,367 O26:H11 strains from 20 countries and revealed a new genetic clade, designated ST29C4. We subsequently evaluated the potential of the already-existing typing technology for identifying new O26 clones, including MLST, PGP, and CRISPR typing, and further compared the differences of multiple genetic elements among O26 lineages.

## RESULTS

### Whole-genome-based phylogenetic analysis of Escherichia coli O26:H11 strains.

**FIG 1:**
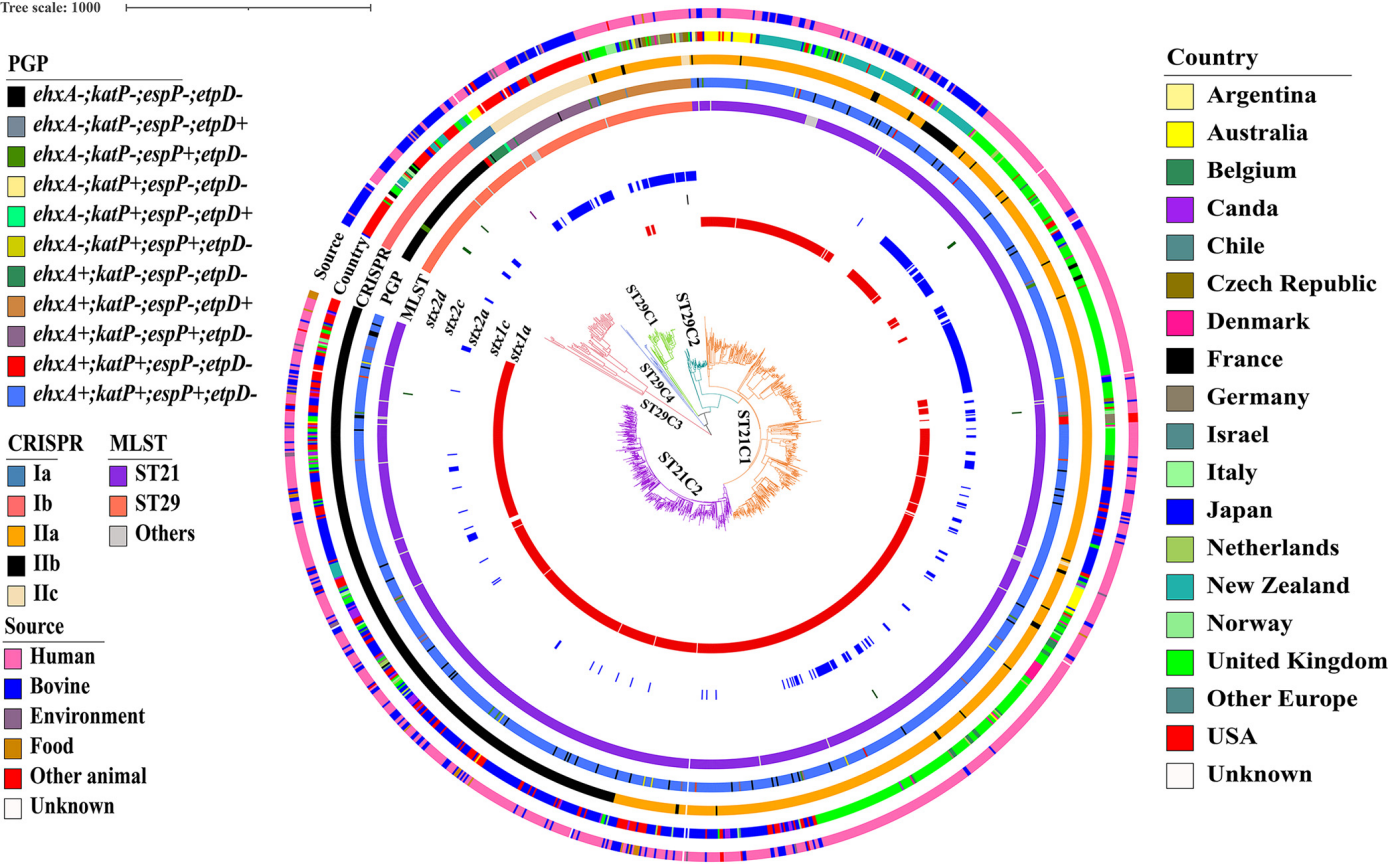
The WG-based ML phylogenetic tree of 1,367 O26 strains. From inside to outside, the colored rings indicate the *stx1a*, *stx1c*, *stx2a*, stx2c, *stx2d*, MLST, plasmid gene profile (PGP), CRISPR subgroup, country of isolation, and source of isolation, respectively.

The internal *in silico* whole-genome sequence (WGS) quality control analysis demonstrated that all 1,367 strains used in this study were positive for the *wzx_O26_* or *wzy_O26_* genes and harbored the *fliC* H11 allele. These strains were distributed in 20 countries (Table 1 and Fig. 1). Most strains were from UK (*n* = 386), Japan (*n* = 328), USA (*n* = 275), New Zealand (*n* = 111), or Australia (*n* =53) (Table 1). Of them, 980 and 320 strains were human and bovine isolates, respectively. *In silico* MLST analysis showed that most isolates were ST21 (1115/1367, 81.57%) or ST29 (213/1367, 15.58%), and a small number of isolates were the variants of ST21 (30/1367, 2.19%) or ST29 (9/1367, 0.66%) (Table 2).

**TABLE 1:** Summary of 1,367 Escherichia coli O26 strains analyzed in this study

Country	Host						Year of isolation
	Human	Bovine	Other animals	Food	Environment	Unknown	
Argentina (*n* = 2)	2	0	0	0	0	0	1999-2002
Australia (*n* = 53)	52	1	0	0	0	0	1986-2016
Belgium (*n* = 29)	21	8	0	0	0	0	1984-2013
Canada (*n* = 37)	13	11	0	0	9	4	1977-2011
Chile (*n* = 1)	0	1	0	0	0	0	2016
Czech Republic (*n* = 16)	16	0	0	0	0	0	2006-2016
Denmark (*n* = 23)	23	0	0	0	0	0	2004-2017
France (*n* = 13)	13	0	0	0	0	0	1999-2019
Germany (*n* = 30)	21	4	5	0	0	0	1996-2017
Italy (*n* = 15)	14	1	0	0	0	0	1992-2015
Japan (*n* = 328)	295	32	1	0	0	0	1994-2016
Netherlands (*n* = 15)	14	1	0	0	0	0	1991-2013
New Zealand (*n* = 111)	32	79	0	0	0	0	1985-2016
Norway (*n* = 18)	17	0	1	0	0	0	2002-2010
Other Europe (*n* = 7)^*a*^	6	0	0	0	1	0	1952-2016
USA (*n* = 275)	57	181	12	17	5	3	1947-2020
UK (*n* = 386)	381	1	0	2	0	2	1967-2020
Unknown country (*n* = 8)	3	0	0	0	1	4	
Total (*n* = 1367)	980	320	20	19	15	13	

a Israel (*n* = 1), Poland (*n* = 1), Romania (*n* = 3), Switzerland (*n* = 2).

**TABLE 2:** MLST types and *stx* profiles of O26 strains

MLST type (ST)	*stx* profile								
	*stx1a*	*stx1c*	*stx2a*	*stx2c*	*stx2d*	*stx1a*+*stx2a*	*stx1a*+*stx2d*	No *stx*	Total
ST21	816	0	112	0	3	134	2	48	1115
SLV of ST21	26	0	2	0	0	1	0	0	29
TLV of ST21	1	0	0	0	0	0	0	0	1
ST29	7	1	94	1	3	0	0	107	213
SLV of ST29	0	0	6	0	0	0	0	3	9

To present a global phylogenetic overview for O26 strains, the ML phylogenetic tree based on 35,668 recombination-free SNP sites on a 3,037,060 bp core genome sequence was constructed. Apart from previously described five lineages (ST29C1-C3 and ST21C1-C2) based on PGP and SNP differences (23), the large-scale phylogenetic analysis identified a new lineage ST29C4 that was formed by 16 strains and separated from other lineages. The pair SNP distances between lineages were higher than those within the same lineage, thereby supporting the lineage designations (Table S2). Moreover, the phylogenetic tree and SNP differences showed that all ST29C4 strains were phylogenetically closer to other lineages (ST29C1-C2 and ST21C1-C2) than ST29C3 (Fig. 1 and Table S1). To better illustrate the phylogenetic relationship of all O26 lineages, we generated an MST using an allele-based core genome MLST (cgMLST) approach. Consistently, all ST29C4 strains were clustered in a distinct branch from other lineages (Fig. S1).

### The evolutionary divergence of O26:H11 lineage is reflected by CRISPR.

**FIG 2:**
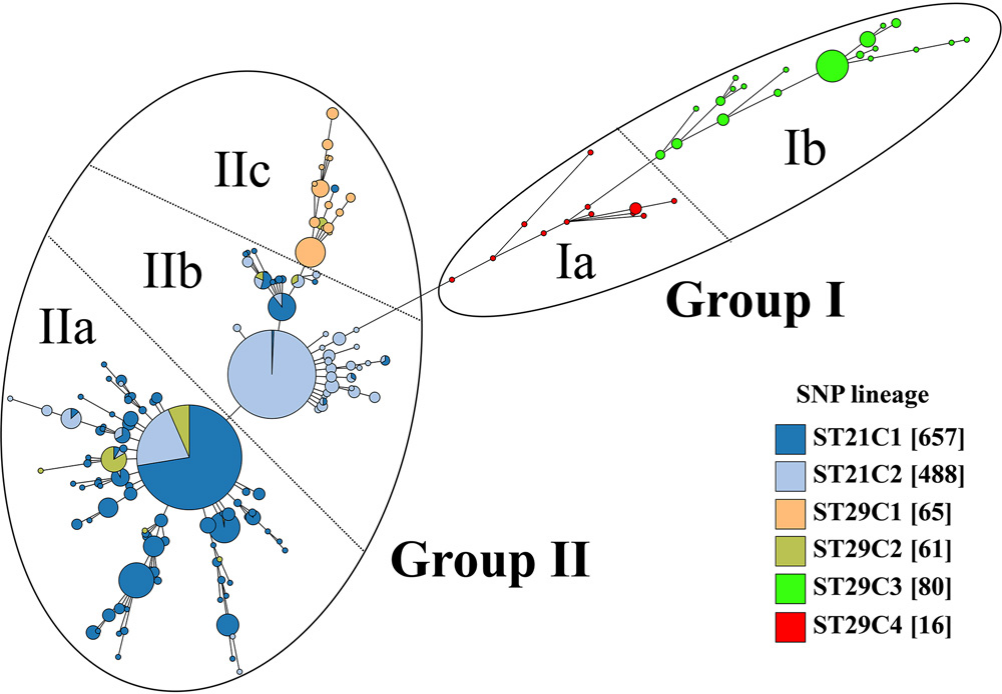
Minimum spanning tree of CTs for 1,367 STEC O26 strains. Each CT is represented by the nodal point, and the number of strains within each CT is indicated by the size of the circle while the relationship among these CTs is indicated by the branch and distribution of all the CTs.

To test whether the evolutionary divergence of O26:H11 is reflected by CRISPR, a minimum spanning tree based on the presence or absence of CRISPR spacer was constructed. As shown in Fig. 2, the MST based on CRISPR spacer showed that all ST29C4 strains were located in the intermediate position between ST29C3 and other lineages (ST21C1-C2 and ST29C1-C2). Moreover, the cluster by CRISPR spacers divided all O26 strains into two main groups (group I and II) (Fig. 2). Group I was further divided into two subgroups (group Ia and Ib), and group II was further separated into three subgroups (group IIa, IIb, and IIc). Interestingly, we observed a high correspondence between the CRISPR cluster and SNP lineage classification. All ST29C3-C4 strains corresponded to group I, and all strains of other lineages corresponded to group II. Further analysis showed all ST29C4 and ST29C3 strains corresponded to group Ia and Ib, respectively, most of the ST29C2 strains (85.24%, 52/61) and most of the ST21C1 strains (92.24%, 606/657) belonged to group IIa, most of the ST21C2 strains (76.64%, 374/488) belonged to group IIb, and all ST29C1 strains corresponded to group IIc (Fig. 1 and 2).

**FIG 3:**
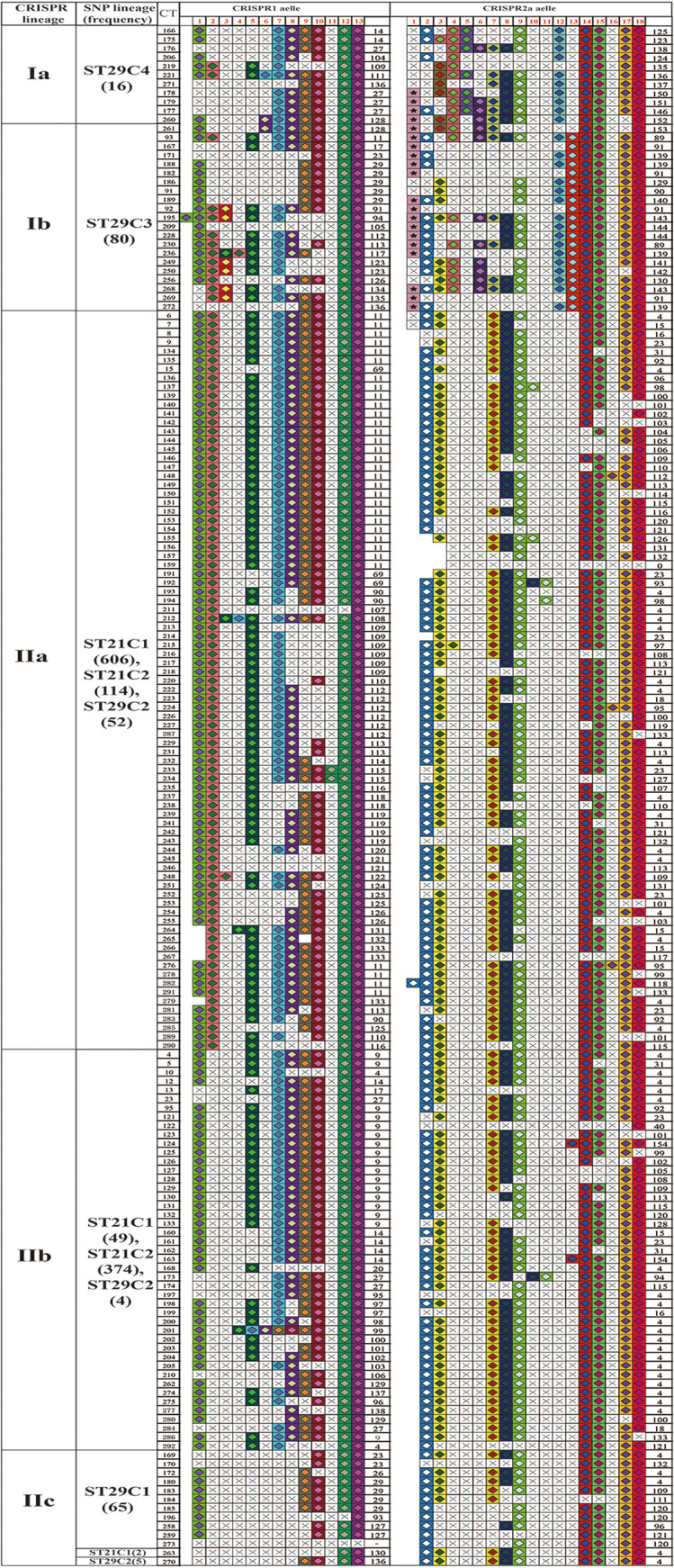
The spacer organizations and arrangements of 1,367 O80 strains. The strains with identical spacer organizations and arrangements were merged. The spacer is represented by one square, and each unique spacer sequence is marked as the combination of a unique color and one center symbol. The symbol in the center indicates the length of the spacer (diamond for 32 bp and star for 1,232 bp). Spacer numbering is initiated at the ancestral end (right) toward the most recently acquired spacers (left) per strain. The left side represents CRISPR1, and the right represents CRISPR2a for the same strain in the same line. The CRISPR lineage, WGS-based SNP lineage, CRISPR type, CRISPR1 allele, and CRISPR2a allele are displayed in the corresponding column.

To further explore the divergence between and within O26 lineages in spacer contents, all O26 strains were subtyped based on the organization of CRISPR spacer. The spacer arrangements of CRISPR1 and CRISPR2a loci in all O26 strains were summarized in Fig. 3. Among them, five strains were negative for the CRISPR1 locus, and one strain was negative for the CRISPR2a locus (Fig. 3 and Table S3). These missing CRISPR loci were designated allele 0 for subsequent analysis. Accordingly, a total of 60 alleles were found in CRISPR1 (4.39% allele diversity, 60/1367), and 68 alleles were found in CRISPR2a (4.97% allele diversity, 68/1367). In combination, the CRISPR1 and CRISPR2a formed a total of 172 CRISPR types (CTs) (Simpson’s diversity index = 0.86; 95% confidence interval (CI) = [0.84, 0.88]).

When further analyzing the spacer difference between groups, all the alleles in group I (ST29C3-C4) differed from those in group II (ST29C1-C2, ST21C1-C2) by the spacers at position 4 to 7 and 12 to 13 in CRISPR2a (Fig. 3). The transposon (1,232 bp) that was previously identified (16) was only found in 15 of 24 CRISPR2a alleles belonging to group I. Furthermore, group Ia (ST29C4) and Ib (ST29C3) could be differentiated from each other based on the deletion of the spacer at position 13. Likewise, the strains within group II also showed high diversity in terms of CRISPR spacer contents. Compared with group IIa and IIb, strains in group IIc (all ST29C1 and a small number of ST21C1 and ST29C2) lacked all the spacers at positions 2 to 8 in CRISPR1. Group IIa (most ST29C2 and ST21C1, and a small number of ST21C2) differed from group IIb (most ST21C2 and a small number of ST29C2 and ST21C1) by the spacer at position 2 in CRISPR1.

Accumulating evidence suggested CRISPR could coevolve with associated *cas* genes (24). Thus, all *cas* genes (*cas3*, *cse1*, *cse2*, *cas7*, *cas5*, *cas6, cas1*, and *cas2*) for 1334 strains were concatenated to create a phylogenetic tree to further evaluate the phylogenetic relationship of all O26 lineages. Similar to the result of CRISPR clustering, the phylogenetic tree based on a complete *cas* gene cluster supported the middle location of ST29C4 between ST29C3 and other lineages (Fig. S2).

### The dominated PGP in ST29C4 strains differs from other lineages.

The newly identified ST29C4 lineage was chosen to further characterize the PGP because it had not been the focus in earlier studies. As shown in Fig. 1, ST29C4 strains exhibited three different PGPs. The most common PGP in ST29C4 was *ehxA*+/*katP*-/*espP*-/*etpD* with a positive rate of 75% (12/16). Moreover, this predominant PGP in ST29C4 was occasionally detected in ST21C1 (1 of 657 strains), ST29C1 (1 of 66 strains), or ST29C2 (1 of 61 strains). The second most prevalent PGP in ST29C4 was *ehxA*+/*katP*+/*espP*-/*etpD* with a positive rate of 18.75% (3/16), which was identified in 10 ST21C1 strains. The least frequent PGP (*ehxA-*/*katP-/espP-*/*etpD-*), which was previously recognized as a key characteristic of the new French clone (called ST29C3) (16), was only detected in one single ST29C4 strain. Overall, the dominant PGP in ST29C4 strains is different from other lineages.

### Comparative analysis of *stx2a*-harboring prophages in ST29C4 strains with O104:H4.

We next characterized the *stx* genotypes among O26 strains. Most ST21 strains harbored *stx1a* alone or *stx1a*+*stx2a*, and most ST29 strains were *stx*-negative or contained *stx2a* alone (Fig. 1 and Table 2). Uniquely, one strain, one strain, and eight strains harbored *stx1c*, *stx2c*, and *stx2d*, respectively. Sequence alignment showed that there were two different *stx1a* genes and four different *stx2a* genes among O26 strains (Table 3). The first *stx1a* gene (called *stx1a_1*) was detected in ST21C1, ST21C2, and ST29C2 strains, whereas the other *stx1a* gene (called *stx1a_2*) was specifically detected in ST21C1 strains. As shown in Table 3, most *stx2a* genes also showed an uneven distribution among O26 lineages except that the *stx2a_2* gene was detected in all O26 lineages. A BLASTn search of the *stx2a_2* gene demonstrated that it was identical to that of the O104:H4 strain (strain 2011C-3493, accession number CP003289) that caused the 2011 STEC epidemic in Europe. Notably, the *stx2a* gene sequences in ST29C4 strains were also the same as that of O104:H4 strains.

**TABLE 3:** The distribution of *stx* genes among O26 lineages

*stx* gene	O26 lineage (frequency)
*stx1a_1*	ST21C1 (155), ST21C2 (479), and ST29C2 (7)
*stx1a*_2	ST21C1 (346)
*stx2a_1*	ST21C2 (2)
*stx2a_2*	ST21C1 (18), ST21C2 (9), ST29C1 (2), ST29C2 (48), ST29C3 (8), and ST29C4 (6)
*stx2a_3*	ST21C1 (184), ST21C2 (7), and ST29C2 (1)
*stx2a_4*	ST21C1 (14), ST21C2 (14), and ST29C1 (33)

**FIG 4:**
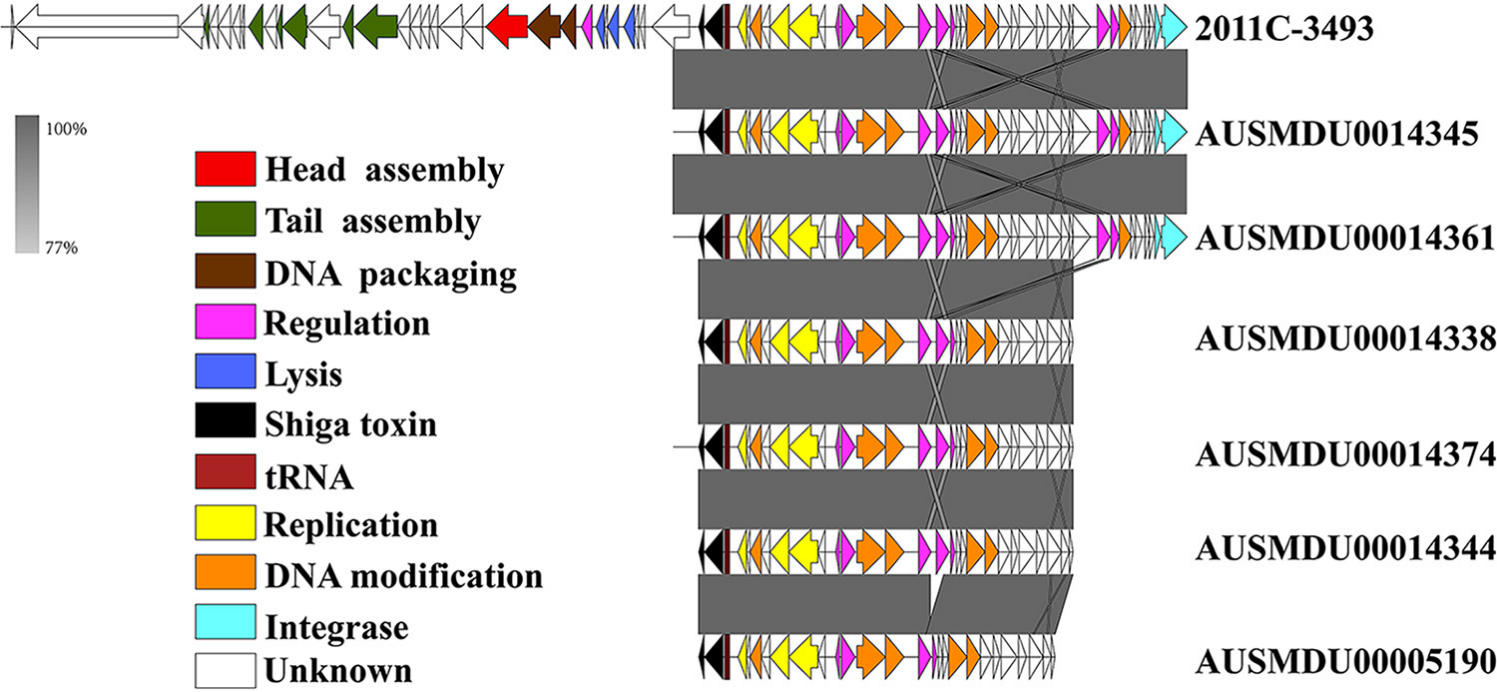
Comparative analysis of *stx*-prophage in ST29C4 with O104:H4 strain. The arrows indicate the open reading frames (ORFs) predicted with PROKKA. The direction of the arrow represents the transcription orientation. The ORFs are color-coded according to their predicted function.

Subsequently, the *stx2a*-harboring prophages in 6 ST29C4 strains and O104:H4 strain (strain 2011C-3493; accession number CP003289) were compared. As shown in Fig. 4, the integrases present in strain AUSMDU00014345 and AUSMDU00014344 had 100% identity with that from O104:H4 strain 2011C-3493. Similar to strain 2011C-3493, the stx2a-prophages in strain AUSMDU00014345 and AUSMDU00014344 were both inserted in the *wrbA* gene. The remaining *stx2a*-prophage regions in the other 4 strains also showed high similarity to that of the O104:H4 strain, although their integrase genes and insertion sites of *stx2a*-prophage in the other 4 strains could be not determined due to truncated genome sequences.

### Diversity of virulence genes and acquired antibiotic resistance genes among O26 lineages.

To further reveal the genetic difference among O26 lineages, we analyzed the prevalence of various virulence genes (VGs). A total of 67 VGs was detected in at least one O26 strain. These VGs detected included genes encoding adhesins (*eae*, *iha*, *lpfA*_O26_, *efa1*, *paa*), type III secretion system effector (35 genes; shown in Table S6), type II secretion system (*gspCDEFGHIJKLM*; referred as to the *gsp* cluster), aerobactin (*iucABCD*, referred as the *iro* cluster), yersiniabactin (*ybtAEPQSTUX* [referred as the ybt cluster], *irp1*, *irp2*, and *fyuA*) and temperature-sensitive hemagglutinin (*tsh*).

All the genes encoding yersiniabactin as well as the *lpfA*_O26_, *efa1*, and *paa* genes were highly conserved in all O26 strains regardless of their lineages. The *eea-β* gene, an indicator of the locus of enterocyte effacement (LEE), was identified in all strains but five strains (25) (Table S1). Apart from these genes, the distribution of some VGs also displayed lineage specificity. The *iha* gene was absent in most ST29C3 strains but present in almost all other lineages. The *iro* cluster was present in ST29C2, ST29C4, ST21C2, and most sublineages of ST21C1. Uniquely, the *gsp* cluster was only detected in ST29C3. Additionally, specific deletions of T3SS effector genes in ST29 lineages were observed although most T3SS effector genes were conserved in all ST21 strains. For instance, the *nleL* and *espN* genes were absent in all strains belonging to ST29C1 and ST29C3, and the *espL1* and *espK* genes were absent in all ST29C3 strains. Unexpectedly, the *tsh* gene was only present in 11 of 16 ST29C4 strains.

We next detected the resistance genes related to 11 classes of antibiotics among all O26 lineages. A total of 531 O26 strains (38.84%, 531/1367) contained at least one antibiotic resistance gene. Also, there was a difference in the numbers of acquired ARGs between O26 lineages. The mean numbers of acquired ARGs were 1.93 in ST21C1, 1.14 in ST21C2, 1.52 in ST29C1, 0.59 in ST29C2, 1.35 in ST29C3, and 0.25 in ST29C4. For ST29C4, only one strain carried resistance genes, including *APH(3′')-Ib*, *APH(6)-Id*, *sul2*, and *tet(C)*.

## DISCUSSION

STEC O26:H11/H− has emerged as the main causative pathogen of HUS, second only to STEC O157 (26). Unlike serogroup O157, STEC O26 constitutes a diverse group and displays a high level of diversity (24). The WGS-based phylogenetic analysis of 27 O26 strains has divided ST29 strains into three genetic clades, designated ST29C1, ST29C2, and ST29C3 (17). Subsequent population structure analysis based on over 500 O26 strains further illustrated that there are at least five clonal lineages in O26 strains (23). In this study, we identified one new genetic clade based on over 1000 O26 strains from 20 countries. According to the previously described nomenclature (23), we proposed to designate the new lineage as ST29C4. The pair SNP distances showed that ST29C4 differed from other lineages by at least 489 core-genome SNPs, which was higher than SNP variation within ST29C4 (Table S2). Compared with other previously established clones, ST29C4 is unique in terms of CRISPR, PGP, and virulence gene compositions (Fig. 1). These findings support the formation of a new lineage, which extends the present knowledge about the clonal structure of O26 strains.

A previous investigation proposed that O26 strains may evolve sequentially from SNP_CC1 to CC4 based on a set of selected 48 SNPs (27). The WG-based phylogeny indicated that SNP_CC1 corresponds to ST29C1 and ST29C3, and SNP_CC2-CC4 corresponds to ST29C2, ST21C1, and ST21C2, respectively (23). However, the phylogenetic relationship between these O26 lineages is still controversial. We found that there were more SNP differences between ST29C3 and other lineages (i.e., ST29C1-C2 and ST21C1-C2) than between ST29C4 and other lineages. This proves that ST29C4 was phylogenetically closer to ST29C1-C2 and ST21C1-C2 than ST29C3 as evidenced by the cluster of CRISPR and *cas* genes (Fig. 2 and Fig. S2). It is thought that highly related E. coli strains are more likely to harbor identical spacer compositions than more distantly related strains (28). In the present study, ST29C4 strains shared similar spacer contents with ST29C3, thereby demonstrating their phylogenetic association (Fig. 2 and 3). The deletion of spacer serves as the main driver of the CRISPR polymorphisms in E. coli and coincidentally correlates with the timeline of strains evolution, which is corroborated in O157 and O80 (29, 30). As shown in Fig. 3, all ST29C4 strains lacked the CRISPR2a spacer at position 13 in comparison with ST29C3. Moreover, the deletion of CRISPR2a spacer between ST29C4 and other non-ST29C3 strains (i.e., ST29C1-C2 and ST21C1-C2) was also observed. Combined with these results, it could be inferred that ST29C4 was located in the intermediate phylogenetic position between ST29C3 and other non-ST29C3 strains (i.e., ST29C1-C2 and ST21C1-C2).

**FIG 5:**
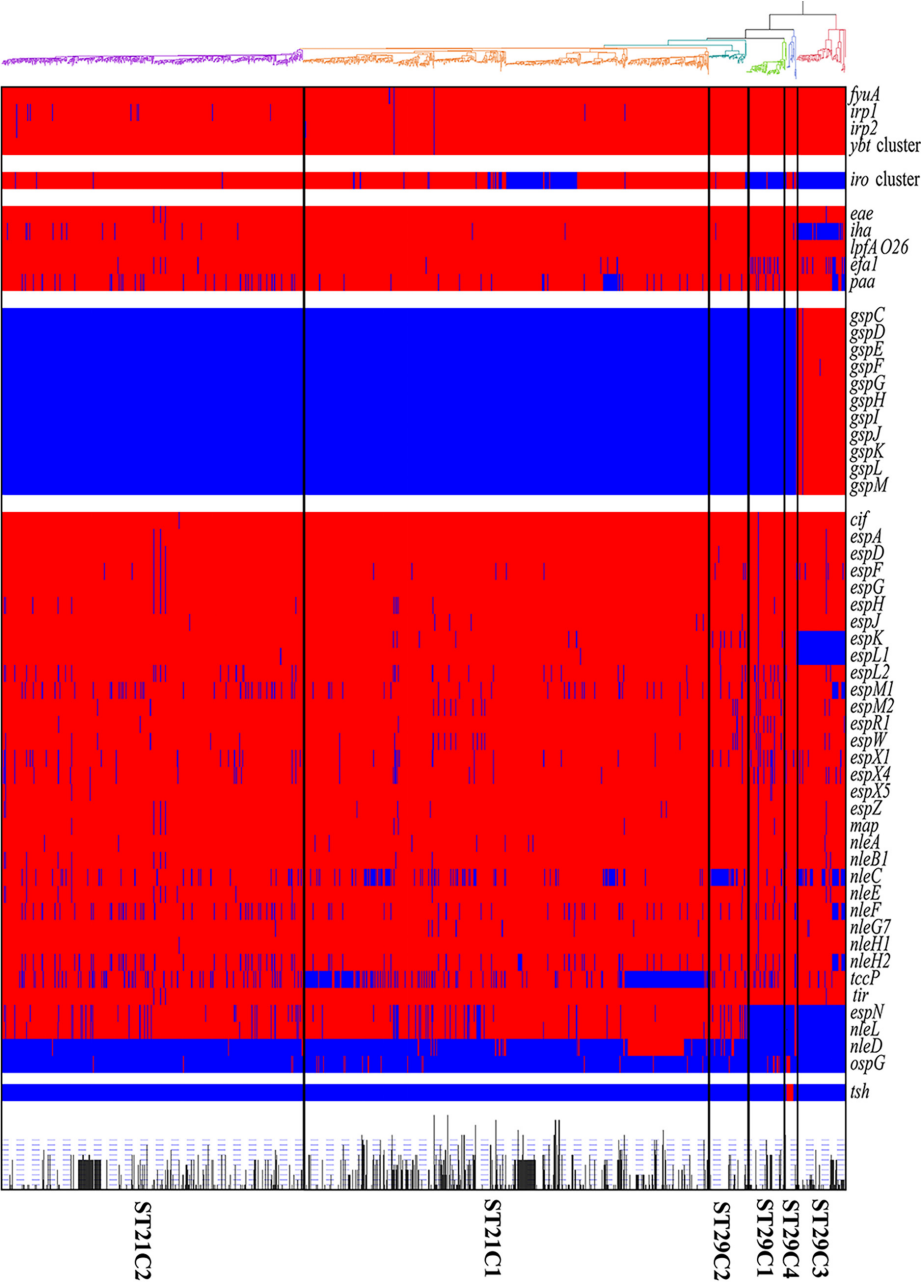
A ML tree of 1,367 O26 strains with heatmaps representing the distribution of 67 virulence genes and the number of acquired AMR genes. The presence of virulence genes is indicated in cell colors: red (presence) and blue (absence). The number of acquired antibiotic resistance is represented by a bar chart.

The distribution of other key genetic elements among O26 lineages also provided new evidence for this evolution mode. The *espK* is ubiquitous in non-ST29C3 strains, conversely, its absence is a key indicator for identifying ST29C3 strains (31). We observed that the *espK* gene was detected in all ST29C4 strains, which reinforced the phylogenetic linkage between ST29C4 and other non-ST29C3 strains (i.e., ST29C1-C2 and ST21C1-C2) (Fig. 5). The complete absence of the *gsp* cluster associated with the type II secretion system in all non-ST29C3 strains, including ST29C4, was another strong evidence for this relationship. The large virulent plasmid (pO157_like) encodes enterohemolysin (*ehxA*), catalase-peroxidase (*katp*), serine protease (*espP*), and type II effector (*etpD*), has been identified in most STEC O26 strains (32). Lineage ST29C3 lacks four plasmid-borne genes, thereby suggesting a complete absence of this plasmid (16, 33). However, the *ehxA* gene indicative of pO157_like plasmid was detected in 15 of 16 ST29C4 strains and 1194 of 1271 other non-ST29C3 strains (i.e., ST29C1-C2 and ST21C1-C2). The other three PGP-related genes (*katp*, *espP*, and *etpD*) were absent in most ST29C4 strains but present in most ST29C1-C2 and ST21C1-C2 strains (Fig. 1). This indicated that a pO157_like plasmid might be acquired by a common ancestor of all non-ST29C3 strains (i.e., ST29C1-C2, ST29C4, and ST21C1-C2) after their separation from ST29C3, and then this plasmid could be stably inherited to ST29C1-C2 and ST21C1-C2 through ST29C4. Overall, these findings further reaffirm the middle location of ST29C4 between ST29C3 and other lineages.

Systemic complication such as HUS induced by STEC infections is largely attributed to Shiga toxin (Stx) encoded by *stx* genes (34). The *stx* genes are carried by *stx*-converting phages and consist of multiple variants, including *stx*_1_ (*stx1a*, *stx1c*, and *stx1d*) and *stx*_2_ (*stx2a* to stx*2k*) (35). It is postulated that enteroaggregative E. coli (EAEC) O104:H4 caused one of the largest foodborne outbreaks in Germany in 2011 through the acquisition of *stx2a*-harboring phage (36). The acquisition of *stx2a* can be considered a general risk factor for HUS development (20). We observed that the *stx*_2_ gene (called *stx2a_2* here, as shown in Table 3) associated with the highly virulent EAEC O104 was detected across all O26 lineages. Not only that, the *stx2a_2* gene was detected in 6 ST29C4 strains from humans. There was a high similarity in *stx2a*-prophage and integrase between ST29C4 and O104:H4 strain, which seems to indicate that this new lineage has the potential to cause HUS. Previous *in vitro* investigation suggested that *stx*-encoding phages from *stx*-positive O26 strains could lysogenize *stx*-negative O26 strains, converting them into STEC strains (14). In this study, we observed that 10 of 16 ST29C4 strains were *stx*-negative. It is possible that these *stx*-negative ST29C4 strains may be the precursor of *stx*-positive ST29C4 strains and can acquire *stx*-encoding phages. Alternatively, they may originate from *stx*-positive strains by the spontaneous loss of *stx*-encoding phages. Further investigation is warranted to substantiate these hypotheses.

Another unexpected finding of ST29C4 strains was that 11 ST29C4 strains possessed the *tsh* gene. Nevertheless, the *tsh* gene was not found in any strain from other lineages, suggesting an independent acquisition event. The *tsh* gene encodes a temperature-sensitive hemagglutinin and is often located on the ColV virulence plasmid in avian pathogenic E. coli (APEC) (37). Besides, the *tsh* determinant is also always involved in urinary tract infection caused by extraintestinal pathogenic E. coli (ExPEC) (38). Here, the coexistence of the *stx2a* and *tsh* genes in six ST29C4 strains was identified (Table S1). The combination of STEC virulence determinants and other pathogroup-defining virulence genes is not rare, contributing to the emergence of hybrid pathogens. Many investigations have elaborated on the prevalence and clinical significance of hybrid E. coli, such as STEC/ETEC, STEC/EAEC, and STEC/UPEC hybrids (36, 39, 40). However, it is the first time that a mixture of STEC and ExPEC virulence factors has been observed in O26 strains, which further reflects the population heterogeneity of O26 strains.

CRISPR can change dynamically in response to exposure of phages or plasmids, which makes CRISPR applicable for bacterial typing and evolutionary investigation (41). In this study, a total of 172 CRISPR types were found in all O26 strains (Fig. 3). According to the results, CRISPR typing could divide strains of the same lineage into smaller units. One investigation in Salmonella typhimurium showed that the discriminatory power of genotyping based on the combination of CRISPR, and other molecular markers were comparable with pulsed-field gel electrophoresis (PFGE) (42). The high resolution will aid in correctly identifying infection sources in short-term outbreaks. Meanwhile, this numerically coded typing scheme also facilitates the exchange and interpretation of typing results between labs. At present, the CRISPR-based typing databases for multiple STEC serogroups have been established by several independent research groups (29–31, 43). Nevertheless, these databases are all based on a small sample of strain collections, which limits the application range of this method. Our current large-scale analysis extends the previous 48 CTs to 172 CTs for O26 strains, which provides a basis for building a global CRISPR typing database for O26 strains (29). The comparative analysis of CRISPR also revealed the conservation of spacer contents and arrangements within lineages. Three CRISPR-targeting qPCR assays have been applied for identifying O26 strains based on spacer conservation (16, 44). Sarah et al. (21) reported that the positive response to SP_O26_E was associated with O26 clones harboring a specific combination of virulence markers. ST29C4 strains analyzed in this study exhibited spacer conservation within a lineage and could be distinguished from other lineages by the difference of specific spacer. Accordingly, CRISPR may be a promising target to diagnose this new lineage.

## CONCLUSION

The WGS-based phylogenetic analysis identified a new genetic clade, designated ST29C4, which displayed genetic uniqueness in terms of MLST, PGP, and CRISPR. Moreover, the combination of multiple typing methods demonstrated that ST29C4 was in the intermediated phylogenetic position between ST29C3 and other lineages (i.e., ST29C1-C2 and ST21C1-C2). Overall, these findings extend our current understanding of O26 strain microevolution.

## MATERIALS AND METHODS

### Bacterial isolates and metadata.

Escherichia coli O26 isolates were retrieved from the National Center for Biotechnology Information (NCBI, https://www.ncbi.nlm.nih.gov/) and EnteroBase databases (http://enterobase.warwick.ac.uk/species/ecoli) (retrieved in November 2020). The read or assembled sequence data of O26:H11 strains were downloaded after confirming the serotyping using the EcOH database. Meanwhile, low-quality sequences were excluded (coverage depth <20×; N50 contig length <40 kb). In some cases, the genome sequence of the same isolate was recorded repeatedly in the two databases. Such duplicates were removed by identifying the biosample accession number. Besides, duplicated strains that harbored identical recombination-free core genome alignment were also removed to construct a nonredundant database (as introduced in the SNP detection and phylogenetic analysis section). Finally, a total of 1,367 O26:H11 strains collected worldwide were included in this study. Specific metadata for each isolate (i.e., geographical origin, host source, and year of isolation) was collected from public databases (Table S1). Among them, 28 isolates lacked at least one of the three above-mentioned metadata. The missing metadata was regarded as unknown. The host sources of isolates were categorized into six groups: human, bovine, other animals, food, environment, and unknown source (Table 1). The raw beef samples were deemed as a bovine source, other nonbovine animal samples (i.e., avian, ovine, and porcine) were categorized into other animals, and nonmeat samples (i.e., spinach, flour, and cilantro) were regarded as food classification.

### SNP detection and phylogenetic analysis.

The chromosome sequence of strain 11368 (accession number AP010953.1) was used as a reference genome for phylogenetic analysis. PHASTER and ISfinder were used to identify the prophage and IS (insertion sequence) region located on the reference genome, and then a phage- and IS-masked reference genome was obtained using Bedtools v2.30 (45, 46). The genome sequence of each isolate was aligned with the phage- and IS-masked reference genome using MUMmer v3.23 (sequence identity ≥98%; alignment length ≥1000bp) (47). The intersection of all the alignment results produced the core genome region that was a length of 3,037,060 bp using our in-house programs (all provided upon request). Subsequently, the genome sequence of each strain was reconstructed using the SNP information present in the core genome. The reconstructed core genome sequences of all strains were realigned by snippy v4.6.0 (https://github.com/tseemann/snippy) and the alignment result was delivered to gubbins v3.0.0 (48). The gubbins software performed recombination analysis and removed 72 recombinogenic sites. Sixteen strains in which the recombination-free core genome alignment was completely identical to that of at least one other strain, were removed for further analysis. Finally, a concatenated alignment of 35668 recombination-free SNP sites on the core genome (3,037,060 bp) was uploaded to RAxML v8.2.12 to construct the maximum likelihood (ML) phylogenetic tree with General Time Reversible (GTR)-GAMMA model of nucleotide substitution and 500 bootstraps (49). The ML tree was displayed by iTOL v5 (50).

### *In silico* cgMLST typing, MSLT typing, *stx* genotyping, *eae* genotyping, and plasmid gene profiling.

*In silico* core genome, MLST (cgMLST) analysis was performed to further infer the phylogenetic relationship of all O26 strains. An E. coli cgMLST scheme comprising 2,513 target loci of E. coli was employed to acquire the cgMLST type based on genomic sequences (22). The minimum spanning tree (MST) was generated by the GrapeTree online tool based on the above cgMLST type (51). *In silico* analysis of MLST was implemented by MLST 2.0 available on the CGE website (identity = 100% and coverage = 100%) using the seven housekeeping genes (i.e, *adk*, *icd*, *fumC*, *purA*, *gyrB*, *mdh*, and *recA*) as queries (https://cge.cbs.dtu.dk/services/MLST/). The reference nucleotide sequences of *stx*_1_, *stx*_2_, and *eae* variants were publicly available and downloaded from GenBank. The *stx* and *eae* genotyping of each strain were determined by *in silico* BLASTn search using *stx* and *eae* reference sequences as queries, respectively (identity ≥ 99% and coverage ≥ 99%) (52). The presence or absence of four genes (*ehxA*, *katP*, *espP*, and *etpD*) related to PGP analysis was determined by VirulenceFinder 2.0 on the CGE webserver (identity ≥ 99% and coverage ≥ 99%) (53).

### CRISPR identifying, typing, visualization, and clustering.

The CRISPR recognition tool was used for identifying CRISPR arrays and extracting spacer sequences (54). The settings were as follows: repeat length 29 nt, spacer length 29 to 34 nt, minimum repeats per array 2. Subsequently, each spacer was compared against the spacer dictionary constructed by previous investigations to acquire the code of the spacers (covered length = 100% and identity = 100%) (29). A new number was assigned for spacers that had no perfect match in the spacer dictionary, and then a CRISPR allele was defined by each unique spacer combination within a CRISPR locus (Table S4 and Table S5). For alleles not previously introduced, a new CRISPR allele number was given. Meanwhile, a CRISPR type (CT) was assigned based on each unique CRISPR1 and −2 alleles combination. The visualization of CRISPR arrays was accomplished by the CRISPRstudio tool (55). The binary library was generated based on the presence or absence of every spacer in CRISPR arrays for each strain. Simply, if a spacer was present in a strain, it was designated “1”. Otherwise, it was designated “0”. The binary patterns of all isolates were submitted to the GrapeTree webserver to establish an MST (51).

### Identification of *cas* genes and phylogenetic analysis.

A previous investigation suggested that one typical type I-E *cas* gene cluster was present in O26 strains (29). The search of type I-E *cas* genes was completed through the local BLASTn tool using strain 11,368 (accession number AP010953.1) as the reference sequence (identity ≥ 85% and coverage ≥ 95%). Type I-E *cas* gene clusters were annotated in strain 11,368 (*cas3*, *cse1*, *cse2*, *cas7*, *cas5*, *cas6*, *cas1* and *cas2* encoded as locus tags ECO26_3831, ECO26_3829, ECO26_3828, ECO26_3827, ECO26_3826, ECO26_3825, ECO26_3824, and ECO26_3823). Once the complete *cas* gene cluster for a strain was determined, all *cas* genes for the strain were concatenated to create an ML tree to estimate the phylogenetic relationship of strains.

### Prophage prediction and *stx*-prophage comparison.

The prophages in strains were predicated using the PHASTER server (46). The determination of *stx*-prophage was implemented through the screening of the *stx* gene in all predicated prophages using BLASTn search. The comparison of *stx*-prophage was performed by EasyFig (56) and BLASTn (52).

### Screening of other virulence genes and acquired antibiotic resistance genes (ARGs).

The presence of other virulence factors and acquired ARGs associated with E. coli were *in silico* screened by the pipeline ABRICATE v0.8.10 (https://github.com/tseemann/abricate) (identity ≥ 95% and coverage ≥ 95%). The pipeline contained known VFs and acquired ARGs for E. coli, which were taken from the VFDB (http://www.mgc.ac.cn/VFs/main.htm) and CARD databases (https://card.mcmaster.ca/).
