# Supplementary material for: Large-Scale Phylogenetic Analysis Reveals a New Genetic Clade among Escherichia coli O26 Strains
Source: Microbiol Spectr. 2022 Feb 2;10(1):e02525-21. doi: 10.1128/spectrum.02525-21 (PMC8809355; doi:10.1128/spectrum.02525-21)
Supplement: SUPPLEMENTAL FILE 1 — Supplemental material. Download SPECTRUM02525-21_Supp_1_seq7.pdf, PDF file, 0.3 MB [file spectrum02525-21_supp_1_seq7.pdf]

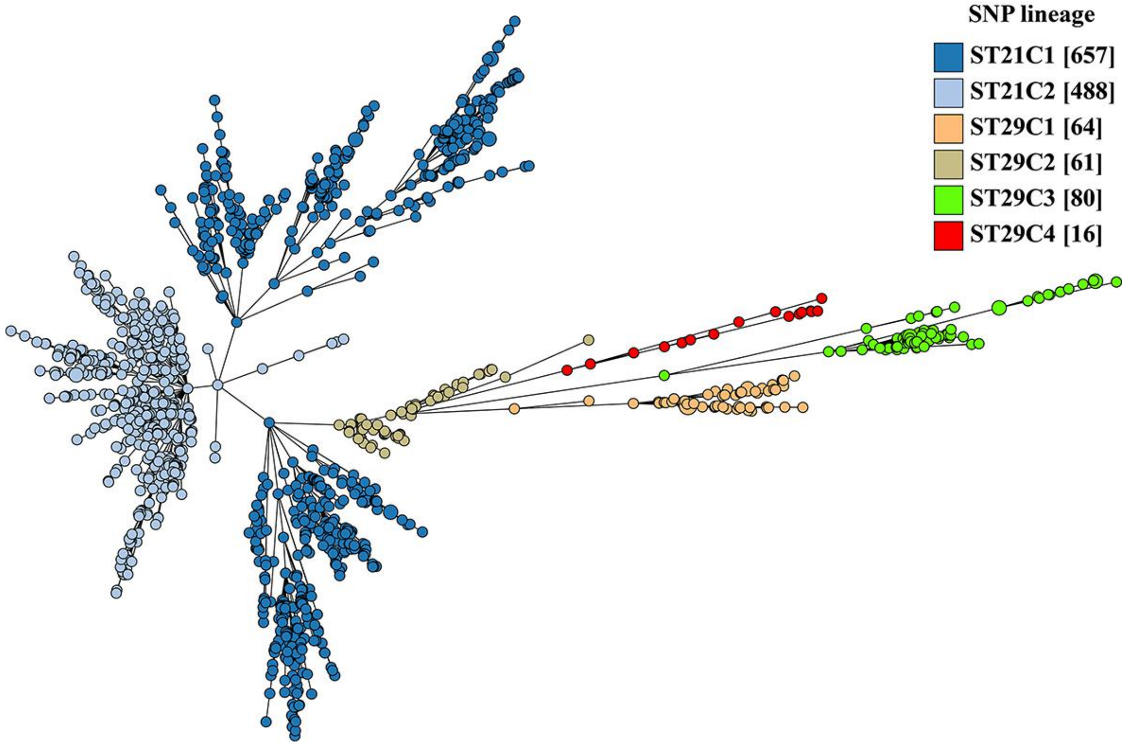

Figure S1 Minimum spanning tree based on cgMLST type for 1367 STEC O26 strains. Each cgMLST type is represented by the nodal point, and the number of strains within each cgMLST type is indicated by the size of the circle, while the relationship among these types is indicated by the branch and distribution.

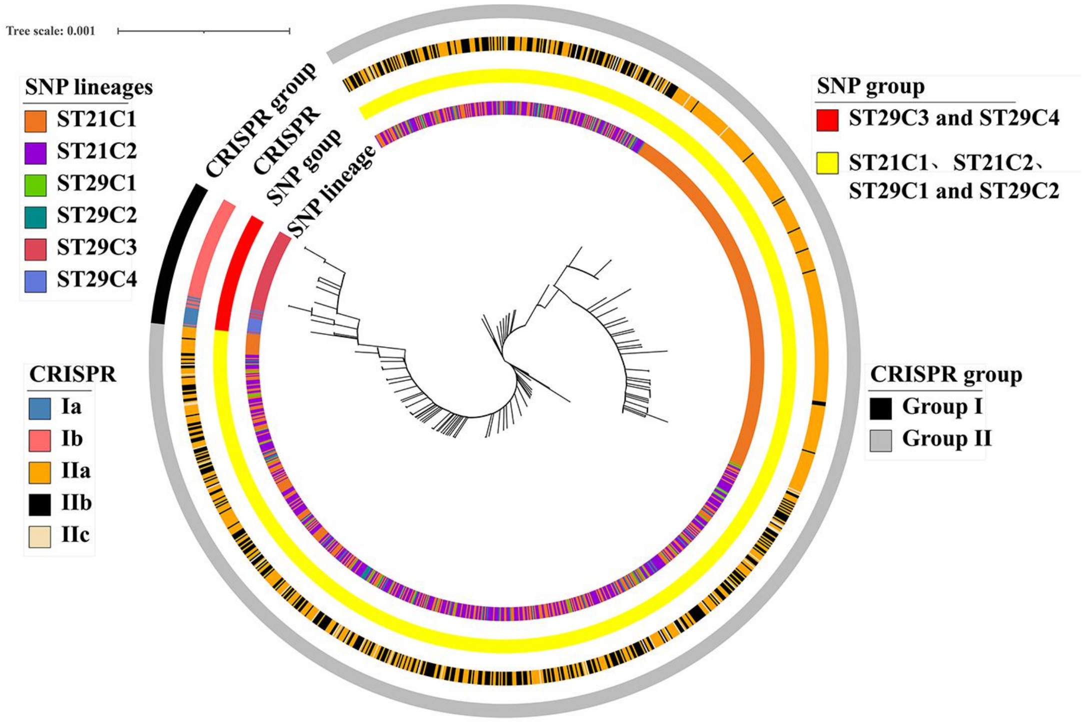

Figure S2 A ML tree of 1344 O26 strains based on seven concatenated *cas* genes. From inside to outside, the colored rings indicate core genome SNP lineage, core genome SNP group, CRISPR subgroup and CRISPR group, respectively.
